# “We Become a Madman to Be Calmed”—Patients’ Voices: Crossing the Threshold of Psychiatric Emergency Departments

**DOI:** 10.3389/fpsyg.2021.709670

**Published:** 2021-07-28

**Authors:** Mathilde Meriaux, Jennifer Denis

**Affiliations:** Department of Systemic and Psychodynamic Clinical Psychology, Faculty of Psychology and Educational Sciences, University of Mons, Mons, Belgium

**Keywords:** welcome, grounded theory methodology, rites of passage, psychiatric emergencies, subjective experience

## Abstract

Crossing the threshold of a psychiatric emergency room is a real ordeal. It is a passage that upsets, worries and sometimes paralyzes. However, it can also become an opportunity if psychological suffering is welcomed, accepted and understood. The welcome is the starting point for care. Our objective is to understand the meaning given to the phenomenon of “being welcomed” by patients experiencing psychiatric emergencies. The research is based on Grounded Theory Methodology (Glaser and Strauss, 1967) to explore and understand the complexity of the phenomenon. The results reveal that being welcomed can be considered as a rite of passage taking place in four successive phases, which are themselves organized into four interactive dimensions. The welcome as a rite of passage constitutes a powerful psychic support which arranges the transitional space in which the individual finds themself, and accompanies the identity transformations, the anguish, and the various sufferings which are not lacking in these moments of crisis.

## Introduction

At present, and in general, there are two ways of accessing mental health care: either by a simple and voluntary approach, which leads the person concerned to request care from an appropriate organization (e.g., private practice, mental health service), or, when the psychological suffering is complex and disconcerting, it becomes a psychiatric emergency. At the same time, the care teams respond to the emergency with immediate availability. In any case, this psychological or psychiatric suffering must be welcomed. This question of how patients are welcomed has been the main theme of most of Guy Baillon and Florence Quartier’s work (Baillon, 1998; Quartier et al., 2015).

Moreover, over the last twenty years, the general emergency world has been confronted with a 150% increase in requests for psychiatric care (Bruffaerts et al., 2006; Denis et al., 2012), especially since the global health crisis we are facing. Although, internationally, the number of patients visiting psychiatric emergency departments decreased significantly during the lockdown, studies show a “psychiatric wave” of the COVID-19 pandemic in the post-lockdown period (Ferrando et al., 2021; Flevaud et al., 2021). Furthermore, the care of psychiatric patients in the general emergency room often refers to a brief examination or is limited to a short interview (Baillon, 2004; Shattell et al., 2014). This interview is more evaluative in order to find the most appropriate orientation for the patient. Thus, specific structures have been created to offer a welcome for this psychological suffering and to develop the care proposals offered to patients. These include psychiatric crisis and emergency departments, the creation of mobile psychiatric teams, crisis hotlines, and psychiatric reception centers. The use of psychiatric emergency departments has particularly increased over the last decades (Schimdt et al., 2020).

In this context, improving care has become a major issue for our institutions, and satisfaction questionnaires have multiplied upon discharge from an emergency department (Gentile et al., 1999; Reboul-Marty et al., 2000; Perruche et al., 2008; O’Regan and Ryan, 2009; Fleury et al., 2019). Complaints are mainly about waiting time, communication by staff and the physical environment. Thus, evaluation, which has become society’s leitmotif, has also gradually become part of our public institutions (Del Rey, 2013). Patient reception has obviously been affected by the many ways of evaluating it. In addition to the satisfaction questionnaires, we can now give a “rating” on the internet and comment on the quality of the welcome and care received. Welcoming patients is now based on the production of figures that are intended to be “objective,” and has been slowly disguised in an administrative and functional form, losing sight of its ethical approach.

We ourselves were caught in this trap when we asked patients about their experiences of being welcomed in psychiatric emergency departments (Meriaux et al., 2019). Far from being based on a quantitative logic, our results reflected the experience of the quality of the welcome received, and not the essence of the phenomenon of being welcomed. Other studies have also paid attention to how patients experienced their care in emergency departments (Allen et al., 2003; Clarke et al., 2014; Harris et al., 2016; Shattell et al., 2014). Although this initial overview seems necessary to understanding the welcome experience, it could now be valuable to conceive of the welcome as a scientific concept that is more appropriately articulated to reality. To this end, its phenomenological meaning must be explored and studied, and this, in its two aspects: welcoming and being welcomed. The first has already been the subject of a publication (Meriaux et al., 2021), so we need to focus on the other side by considering the following research question:

*How do patients make sense of the phenomenon of being welcomed in psychiatric emergency departments?*

## Materials and Methods

The design of this study is based on a qualitative inductive approach because of the nature of the research problem. It is about the experience of individuals (patients) in relation to a phenomenon (being welcomed during a psychiatric emergency).

Grounded Theory Methodology (GTM) seemed to be the most suitable means to answer our research question. This method is suitable for the study of phenomena related to complex social processes and for which there is little knowledge. In such a case, where the problematic leads to the exploration of a social situation, a phenomenon without imposing any hypothesis to be verified and to remain open to what the actors are experiencing, the methodological approach to be favored must not only be qualitative, but it is also preferable that it be inductive (Guillemette, 2006). This methodological approach aims, from empirical data, to produce a substantive theory allowing the understanding of the phenomenon (Glaser and Strauss, 1967). This is in line with our research objective which is to conceptualize the phenomenon of “being welcomed.” Moreover, we chose GTM because it is not just a method of analysis. It is first and foremost an epistemological posture and a general research methodology.

**FIGURE 1:**
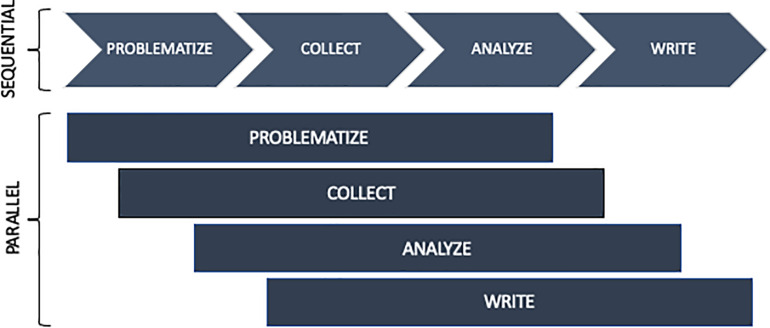
Parallel organization of the research (Lejeune, 2014).

This method is exploratory on the one hand, and inductive on the other. The data from the field are at the center of the approach and actively participate in the elaboration of a theorization. Its purpose is to capture the variability and complexity of the phenomenon (Strauss and Corbin, 1998). The method opts for a “flexible,” “spiral” and “iterative” approach in order to respect two imperative rules: to give a report on empirical material and to create new theories (Lejeune, 2014). More simply, the method begins with the formulation of a research problematization that the researcher will refine during a process in which he collects, encodes and analyzes empirical data. The new data are integrated and confronted as the analysis proceeds. In other words, grounded theory analysis does not begin after the data collection, but at the same time. The research is a constant and progressive back and forth method between the collected data regarding the phenomenon and the theorizing process. Figure 1 illustrates the parallel organization of the research (compared to a sequential organization).

### Ethical Considerations

Before starting the research, we received the agreement of the care management team of the different psychiatric emergency services, as well as a favorable opinion from the ethics committee of each hospital. Confidentiality was maintained throughout the research process and each participant gave free and informed consent prior to their inclusion in the study, in accordance with the obligations of the General Data Protection Regulation (GDPR).

In addition, our interviews concerned clinical situations prior to the investigation. More concretely, we only retained patients referred to crisis beds within psychiatric emergency services in order to avoid altering and burdening the initial care with an additional interview in the psychiatric emergency department. Our research process is intended to be respectful of the integrity of the participants, non-intrusive and in accordance with the clinical reality of health professionals and patients in psychological suffering.

### Data Collection

#### Sampling and Theoretical Saturation

The theoretical sample was determined by the following research question: How do patients make sense of the phenomenon of being welcomed in psychiatric emergency departments? Therefore, it was formed by selecting patients who had received urgent psychiatric interventions and were hospitalized in a crisis unit. Participants were selected because of their theoretical relevance to the research process, not based on their representativeness to the population of interest (Strauss and Corbin, 1998). Participants had to be of legal age (18 years old), and the proposal to participate was based on a clinical assessment by a team member, evaluating the patient’s state of anxiety and the absence of behavioral disorders. By team members we mean: psychiatric nurses, psychiatrists and psychologists. It should be noted that in qualitative research, we are looking for variability in experiences. Our goal is not to count.

Data collection was conducted in three psychiatric emergency departments with crisis beds between January 2019 and January 2021. Two of these are located in Belgium, and the third in the north of France. The structural and spatial-temporal organization is somewhat different in each of these services. One is located within the general emergency department (which we will call place A). For the other two sites, the psychiatric emergency department is delocalized from the general emergency department, and comprises two geographically contiguous poles: the psychiatric reception unit and the crisis unit. These units only receive requests for psychiatric care and are not involved in somatic care (location B).

The iterative nature of the method led us to select each of our participants following a series of two interviews. With each direct analysis conducted, we considered the patient’s characteristics, the demand, and the mode of arrival at the emergency department to strengthen our theoretical sampling. Our theoretical sample was gradually built up through the 13 participants. Eleven of them were from psychiatric emergency departments (Table 1).

**TABLE 1:** Characteristics of participants.

	Gender	Age	Characteristics of the request	Place	1st time?	Arrival mode	Accompanying person/sender	Voluntary or involuntary hospitalization
P01	F	24	Suicide attempt and Romantic breakup	A^a^	Yes	Car	Mother/Physician	Voluntary
P02	F	18	- Family problems—Active suicidal ideation	A	No	Ambulance	Alone/General emergencies	Voluntary
P03	M	51	- Romantic breakup - Family problems - Drug intoxication	B^b^	Yes	Car	Alone/Physician	Voluntary
P04	M	41	- Difficult bereavement - Confused thoughts	B	No	Public transport	Alone/voluntarily	Voluntary
P05	F	29	Suicide attempt (suspicion of bipolar disorder)	B	Yes	Ambulance	Mother/General emergencies	Voluntary
P06	F	35	Alcohol detox	B	Yes	Car	Alone/voluntarily	Voluntary
P07	F	27	Active suicidal ideation	B	Yes	Car	Mother/Mother	Voluntary
P08	F	47	Major depression, suicidal ideation	B	Yes	Car	Husband/voluntarily	Voluntary
P09	F	31	Episode of delirium	B	No	Car	Mother/family environment	Involuntary
P10 CC^c^	M	22	Motorcycle accident—Somatic emergencies		No	Firefighters	Parents/firefighters	Involuntary
P11	F	25	Psychotic episode	A	No	Police and firefighters	Alone/police	Involuntary
P12 CC	F	25	Travel Home—Australia, Indigenous Peoples of Latin America					/
P13	F	44	Suicide attempt	A	No	Ambulance	Alone/General emergencies	Involuntary

**^a^Psychiatric emergency room located within the general emergency department. ^b^Psychiatric emergency department separate from the general emergency department. ^c^CC, contrasted cases.**

### Semi-Structured Interviews

Through semi-structured interviews, we invited patients to narrate their experience of the welcome they received. By welcome process, we mean the initial contact with institutions and team members (see above). The semi-directed interviews were conducted during this short hospitalization in the crisis unit, a few days after their care in the psychiatric emergency unit.

The framework for conducting the interviews initially included five to eight key words to guide the investigation. Pragmatically, keywords were inserted into the questions and were intended to gather descriptive, analytical, reflective, and theoretical information (Denis, 2016). The questions focused on the subjective experience at each stage of care, the antecedent (arrival mode, demand, psychological state, expectations and representations), the impact (positive and negative) directly observed inside, and the interactions in the waiting room and during the clinical consultation.

Questions focused on the “how” were favored: How did you feel before, during and after the first clinical situation? How was that first contact? How did you deal with your anger/anxiety/fear? In keeping with the principle of continuous comparison between categories and the raw data, the keywords evolved over the course of the research process (Lejeune, 2014). All the interviews were recorded in audio format in order to facilitate verbatim transcription and, above all, analysis.

Here is a table (Table 2) that will allow the reader to understand what kind of information we wanted to collect according to the nature of the keywords.

**TABLE 2:** Types of keywords in the interview guide.

Descriptive	Analytic	Reflexive	Theoretical
Keywords oriented on the context of arrival, the care process and prior representations of psychiatric emergencies	Keywords oriented to the patient’s experience of the welcoming phenomenon at different moments of the process	Keywords that help the patient to critically reflect on his experience and to see it differently	Keywords that aim to collect useful suggestions or recommendations related to the welcome.
(e.g., First impressions; expectations and representations of emergencies; atmosphere)	(e.g., experiences before, during and after the first encounter; waiting; type of relationship)	(e.g., expectations confirmed? important aspects; if it were to be done again?)	(e.g., Need? Missing? appreciated? improvements?)

### Data Analysis

**FIGURE 2:**
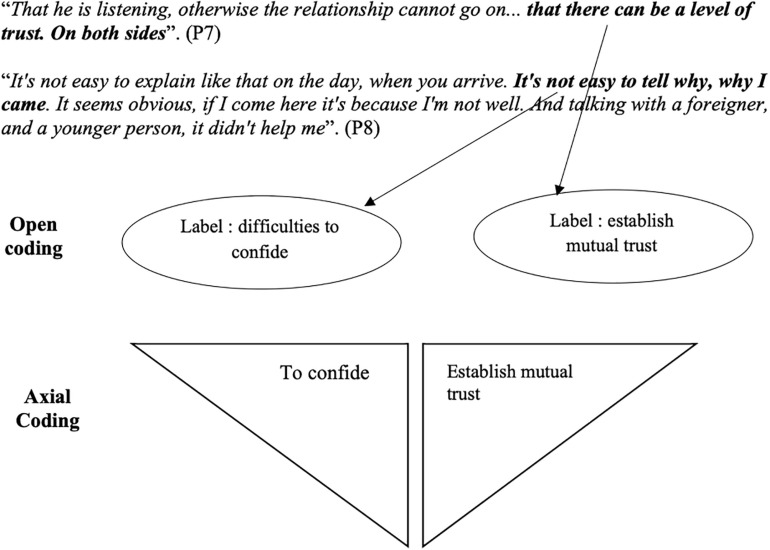
Example of the transition from open coding to axial coding.

In order to achieve the definition of emergent theory, Strauss and Corbin (1998) propose a coding process with three levels of analysis: open coding, axial coding and selective coding. Open coding, the first step in the coding procedure, allows the researcher to label the empirical data. The objective is to assign a word or a group of words to what the participants say about the phenomenon. The researcher stays true to the discourse (Urquhart, 2013) and draws meaning from it. Indeed, we used the verbatim transcript to develop a code based on *in vivo* words. These words translated the expression of an emotion, an affect or a lived experience. The progressive coding of these interviews was carried out under the supervision of qualitative researchers: our thesis director and an expert and trainer in qualitative researches to ensure researchers triangulation. Next comes the axial coding, which is carried out in bursts of two interviews. At this stage, we ask ourselves questions such as “which codes are related?”; “how do they vary together?” Very quickly, these questions made it possible to perceive the variations of a code depending on the situation. The diagrams facilitated this work of articulation and connection. These codes are likely to become “properties” of a theoretical category. The properties are articulated two by two in order to bring out main categories and sub-categories. Axial coding allows properties to be prioritized and assembled, categories to be distinguished from subcategories, and properties or categories to be linked together and varied according to their similarities or dissimilarities. The last step, selective coding, consists of identifying one or more central categories, organizing them, deepening them and developing an illustrative model of the phenomenon studied. To illustrate the passage from open coding to axial coding, we propose, below, Figure 2 which shows an example of this coding system used in our research process.

The first researcher interviewed all of the participants, transcribed the material, and conducted the analysis. The analyses were presented and submitted to two qualitative researchers in order to distance itself from the study field (the thesis director and a qualitative research trainer). This triangulation of researchers ensures the credibility of the research (Gohier, 2004). The traces of this analysis process are recorded in the logbook, which remains the property of the researcher. This logbook includes all the coding reports, theoretical reports, and operational reports that led to the final theorization.

## Results

The in-depth and comparative analysis of our dataset led to an understanding of the essence of the phenomenon of being welcomed. “Being welcomed” is a process that is articulated within a precise temporal sequence that marks the crossing of a threshold, and is distinguished through four successive phases. Each of these is organized according to four interconnected dimensions: the atmosphere, the beliefs, the emotional and perceptive experience and the intersubjective relational experience.

**FIGURE 3:**
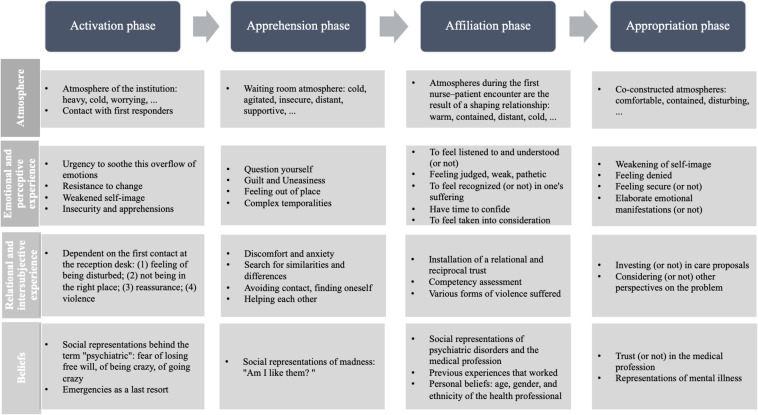
Modeling the phenomenon of “being welcomed.”

We present all of our results using an explanatory diagram (Figure 3). The third phase, which is decisive in this experience of welcoming, will be described in more detail.

The first phase responds to the individual’s and/or their system’s desire for change in the face of the context of suffering, vulnerability, or even despair. Where this context, although costly in terms of resources, was previously manageable, the emotional outburst shows an experience that escapes the control of the usually mobilized defenses. The possibilities are shrinking. Continuing in the same way is no longer possible. A third party is called upon, either voluntarily or involuntarily. This third person can be a relative, a healthcare professional, or the emergency department. In all cases, a process of “activation” is triggered and manifests itself on the perceptual, cognitive behavioral and affective levels. The state of imbalance in which the individual and their system find themselves pushes them into action. There is a reactivation in order to find a new homeostasis. This activation process continues until the arrival at the psychiatric emergency department. Figure 3 gives an overview of what is at stake at the level of the atmosphere, emotions and perceptions, relational experience, and beliefs. These dimensions intertwine and organize the first phase of the process of being welcomed.

*”I need to find solutions to live.” (P3)*

The second phase is characterized by apprehension, presented as an obligatory passage and one which places our participants in an intermediate state between agitation and pause, an uncomfortable in-between in which their bearings are swept away. Participants are disconnected from their environment and from their daily flow of activities, they are busy questioning their norms, their values, their rules, their behaviors. This stage can be very trying. This “time off” forces them to think and dwell on what brought them here, to worry about the before, during and after. The thoughts are ambiguous because they are made of both hopes and fears.

*“to dwell on everything that had happened: why I was there, what I was going to do, what were they going to do to me, what was going to happen to me, was I going to get out of it, was it going to get worse, was the medication any good? All that kind of stuff.” (P3)*

The “affiliation” phase takes shape during the first encounter with the emergency team (i.e., psychiatric nurses, triage nurses, psychiatrists, psychologists, receptionists). It is characterized by the search for supportive links, links that will allow individuals to confide in others. This approach is difficult because it involves the person’s private life, and it will only be possible if the person feels sufficiently confident during the interview. This trust is the result of a dynamic intersubjective process that involves different variables.

*“It’s not easy to explain on the day, when you arrive. It’s not easy to tell why, why I came. It seems obvious, if I come here it’s because I’m not well. And talking, especially with a stranger, and someone younger than me, didn’t help me.” (P8)*

The participants “continuously” test the trust they have in the institution and its actors. Their trust is based on the specificities of the emergency department—the atmosphere they feel, the architecture and cleanliness of the place—and on the specificities of the care relationship. Some atmospheres envelop and form a reassuring cocoon thanks to the attitudes of the health professionals: a smile, a look, a soft and calm voice. The participants pay attention to the presence or absence of smiles, an indicator that strongly orients what appears to be the warmth of the atmosphere in the encounters. They are also very sensitive to the gazes. Different types of gazes were perceived: worried, astonished, curious, severe, cold, strange, inquisitive, benevolent, or even haughty. The use of some words allows a warmer contact to be created. It is a relational trust that relies on the relational qualities of the professional.

*”Yes, by her personality, her voice, [.]a soft voice like that [.]always looking in the eyes, with a smile. she was very nice. Like you do now because you do it too. You can see in people’s eyes: where there is hatred, contempt, or else compassion.” (P3)*

In a second step, the participants assessed the competence of the professional in carrying out the care. They relied primarily on the position held by the health professional—and therefore on their qualifications. Then a more detailed evaluation was carried out during the consultation. The participants paid attention to the voice of the professional, its intonation, its volume, its flow: Did they stammer? Did they search for their words? Did they hesitate? Seem stressed? Worried? They also paid attention to the clarity and transparency of the information provided. Trust is built when the partner provides explicit, two-way information: the concerns and uncertainties of the people met are reduced, they feel like partners in the relationship and see that their opinion is taken into consideration.

Then, the personal characteristics of the professionals influenced the construction of the bond of trust: age, gender, ethnic origin. Some participants noted the difficulty of confiding in someone younger than themselves; they did not doubt their competence, but their life experience, their ability to contain anxieties and understand suffering. Trust is also based on personal beliefs and past experiences.

*“I said to myself ‘he is the same age as me’. It’s more complicated to confide in someone who is maybe younger.”* (P6)

Of course, trust in the caregiver is not just a matter of competence. The relational component naturally brings out this confidence and can even counterbalance the initial prejudices. The final point that is essential to building this bond of trust is that of reciprocity. Patients need caregivers to trust them *”I need there to be a level of trust. On both sides.”* (P6)

*“It’s important that people trust me because it means that they believe me when I say things. Because I often had the impression that the physicians did not believe me and made me repeat things: ‘Are you sure that’s it?”’* (P1)

Trust is a bilateral postulate of listening and benevolence toward the other, thus feeling allowed to describe oneself, to confide and open up to others and to be oneself in the relationship, without fear of being judged. But this trust is not always there. Worse, it can already be tainted when the demand for care is not voluntary. In these situations, the participants involved were not very supportive of the proposed listening, as the following verbatim shows us:

*“we are not very open to care since we do not want to be there. As a result, we don’t want to talk, to share ourselves, to accept treatment because the link doesn’t exist and we don’t have confidence in the caregivers, in the rest of our care, in our future.” (P13)*

For these people, taking time is the best way to gain their trust.

When this trust cannot be established, participants share their reluctance to confide in us. Painful feelings dominated: “*I don’t know. I cried, I felt ridiculous [.] I felt weaker and more pathetic.”* (P7)

One of the reasons mentioned was the establishment of symmetrical escalations in the emergency room where everyone tries to have a dominant position. For one of our participants, being considered “a crazy person to be calmed down” canceled any possibility of creating a bond of trust that would allow her to tell her story.

*“On the side of the caregivers, I think that I was first taken in by “general” emergency professionals without specific training in the care of psychological disorders. I believe that they are afraid that we put ourselves in danger, that we put others in danger, that we escape or whatever. And our word is immediately questioned because we are “in crisis.” So we no longer exist as a person with emotions and thoughts, we become ‘a madman to be calmed’. From this comes infantilization, threats, restraint. This reinforces our opposition: ‘I am forced, therefore I resist’ and makes care even more difficult. In fact, the relationship is not created and this is painful for everyone, I think.” (P13)*

This testimony reveals the various forms of violence suffered in the emergency room. Physical restraint is experienced as a real trauma, which may mark people for life and leave traces that are sometimes irreversible.

This affiliation phase also depends on how well the participants feel they are listening. Feeling listened to is a positive experience that manifests itself in the perceived level of attention, time and availability of the caregiver. These three elements are intrinsically linked. According to our participants, when the health professional takes the time, they send a strong signal that all of their attention is focused on the other person, on their experience and history. It is not just a matter of the caregiver giving time, “mathematical” time, counted and objectified. What the participants want is to be in the presence of someone who listens, in the here and now.

*“Someone who gives you time to express yourself, who doesn’t look at you taking notes and say ok ‘we’ll prescribe you a medication, goodbye, thank you, see you tomorrow’. It’s staff that listens, it’s staff that is present. smiling again. For me, smiling is very important, and especially available.” (P6)*

According to our participants, they appreciated having time to sit down, think, cry, hesitate, without perceiving signs of irritation from the caregiver.

Finally, feeling listened to depends on the caregiver’s consideration for the patient. One participant identifies the effect on her of taking into account her opinion, her needs, her agreement:

“*Yes I felt very judged. In fact, I feel like I wasn’t taken into account as a whole person. That here, they speak to me, they asked me if I wanted my mother to come. They asked me if I was pleased with the suite they were offering, and again this morning when they called me to come, they asked me if I still wanted to come.” (P1)*

Nevertheless, participants may feel that the other is listening, but not listening well. Patients describe the pain they felt when they felt that the caregivers were labeling them as an alcoholic, a bad mother, weak, etc.

“*But there’s one thing I still don’t admit, but okay. It’ll pass [.] that they say I’m an alcoholic. I’m not an alcoholic. It’s just that I took some medication with alcohol, that’s all [.] Moreover, when the nurse came to me ‘it’s probably due to the alcohol that you’re shaking, we’ll give you some medication’. I don’t know how many times I heard that.” (P7)*

*“I felt like the last of the bad moms.” (P6)*

Fear of being judged or lectured was one reason why participants were tempted to withhold information. They were afraid of being seen as “bad people.” Patients’ reluctance to confide comes from the fear of being judged: “*the previous times I’ve come to the emergency department for psychological problems, I always lied because I didn’t feel comfortable telling them things*.” (P1)

According to the participants, feeling judged depends on several factors:

▪ the attitude of the health professional,
▪ the repetition of certain questions that lead to the feeling of not being believed,
▪ note-taking,
▪ an inquisitive gaze,
▪ hurtful words,
▪ the time given,
▪ judging oneself.

What is experienced during this process will partly influence the last phase of this process, called the appropriation phase. When the experience has been positive, it has helped to restart a blocked process, to revitalize a frozen system, and to consider other understandings, other possibilities. Movement is now possible. This new status of the patient, the fruit of a relational shaping, thus brings comfort, security and a certain dose of containment: “*And that reassured me. I felt taken care of*” (P5).

But even if this experience was positive, social representations are still active. Having care in a psychiatric emergency department can weaken one’s self-image, destabilize one’s reference points, and make one uncomfortable, sometimes even ashamed:

”*it’s complicated to go to the other side of the fence. I have a hard time with it, I still have a hard time with it now, but I need it, so I ignore it.*” (P6)

When the experience was violent, the participants had difficulty investing in the care proposals. They were described by the team as “withdrawn,” “refractory,” and “in non-demand.” These people named the wounds caused by this violence and testified to the impossibility of putting their emotions, their experiences, their suffering and their needs into words. In fact, the emotional manifestations could not be elaborated or symbolized. They were still active. For one of the participant (P13), trust in care, and in those who provide it, was not yet conceivable, even to the point of refusing any kind of help, sometimes for years. The following extract testifies to the consequences of the violence experienced during psychiatric emergencies:

”*I experienced it as an injustice, an abuse of power, the negation of what I am, as if they wanted to silence me, to immobilize me because I was disturbing. In fact, it awakened many negative feelings that prevented any honest exchange afterwards. It really broke my trust in the medical profession, and in turn, my care and my health care pathway.*” (P13)

## Discussion

### Being Welcomed as a Rite of Passage

**FIGURE 4:**
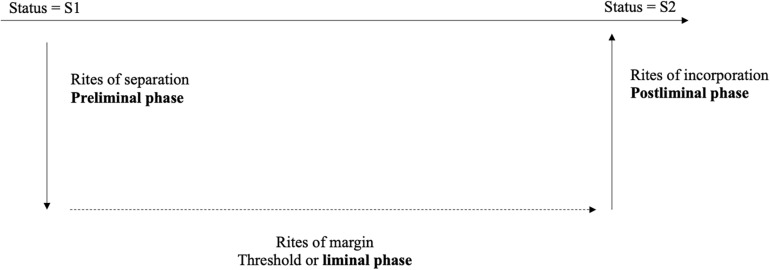
A schematic representation of rites of passage based on Van Gennep (1909) and Turner (1966).

Although emergency departments are considered the gateway to psychiatric care, it is often at this stage that people encounter the world of psychiatry or mental health for the first time. This passage toward “soon-to-be-patient” is marked by certain difficulties. It worries, it agitates, it upsets. It is following this analysis that the question of the “status passage,” approached by B. Glaser and A. Strauss (1971), seemed relevant to us. These authors define it as a set of transitional social processes marking the passage from one status to another. This transition generally involves a change in identity and behavior. From there, we directed our research toward the work of Van Gennep (1909), who approaches “rites of passage” in his work and raises the question of the statutory transition. The French folklorist and ethnologist (1909) underlines that the individual, even a Westerner, recognizes the life stages which punctuate their changes of age, of social situation, as well as changes to their place of dwelling or work. Each of these important modifications is accompanied by rites, which he named *rites of passage*. The rites of passage prepare or accompany the passage by a person from one state to another of their existence. Van Gennep (1909) thus analyzed the rites that punctuate the phases of the life cycle and distinguished three successive stages which are the rites of separation (from the old environment), the rites of margin, then the rites of incorporation (into the new environment). Rites that could also be analyzed as preliminary rites (before the threshold), liminary rites (on the threshold, in between) and post-liminary rites (after the threshold). The process envisaged by Van Gennep, and refined by Turner (1966), is presented schematically in Figure 4.

All the rites of passage are thus articulated around precise times that frame the crossing of a threshold. This symbolic threshold represents the physical and psychic threshold that the person must cross to pass from one state to another, and from one group to another. This threshold is easily identifiable in the context of our study since the place of welcome is the threshold (of the door), the limen-liminis (threshold, limit). “Being welcomed” is thus a matter of passage. The person who “welcomes” does not cross this limit but accompanies the person who crosses it. This passage probably induces the transformation of a stranger integrating a new (social) scene into the protagonist of this scene. They become a “soon-to-be-patient.”

Van Gennep (1909), followed by other authors, such as Gotman (2001) and Boudou (2012), already assumed that hospitality procedures could be assimilated to rites of passage. These allow for the regulation of the incorporation and separation of the stranger within the group. For Van Gennep (1909), the rituals of hospitality strongly mark the precaution with which the hosts think about the arrival of the stranger. This is done in order to erase, as much as possible, the hostile character that the stranger’s presence may imply (Montandon, 2008). This is how Van Gennep (1909) identifies, in his anthropological studies, different rites of hospitality between foreigners and natives. A series of codes are at play in order to condition the success of the encounter.

In our case, the reception is part of a clinical component where what is at stake is to facilitate this soon-to-be-patient and to create the outline of a link which can allow engagement in care.

Van Gennep’s conception has already been recast from a clinical perspective: family, institutional, network (White, 1986; Dessoy, 1997; Courtois, 2003). For example, Dessoy (1997), and later Gelin et al. (2014), propose a conceptualization of individual, couple’s, family, and institutional therapy as rite of passage processes. Here, therapeutic change is the goal. But for this to happen, another rite of passage must first be considered, which both precedes it and will determine its conditions: the welcome between the patient and the practitioner. The future of the therapeutic follow-up depends on it. This first step is costly and insecure, and if the person in pain is uncomfortable, there is a risk that a relationship that has barely begun will be interrupted or that it will continue under unsettled conditions.

### Toward a Model of the “Welcomed Being”

**FIGURE 5:**
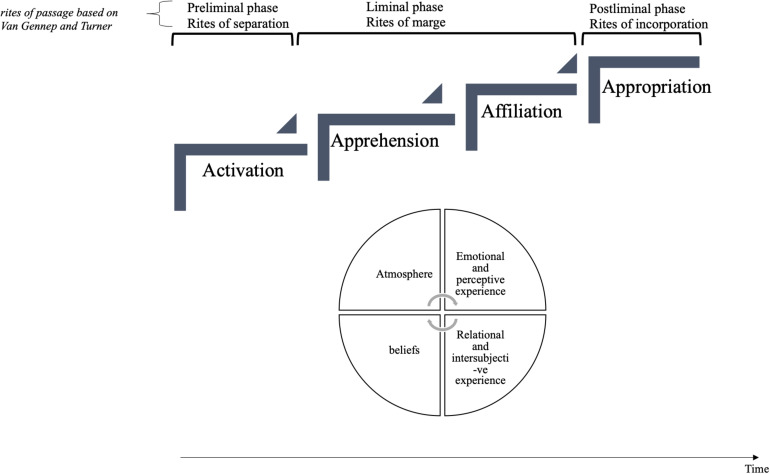
Being welcomed as a rite of passage.

We consider that the way of being welcomed represents this space-time allowing the stranger to cross the threshold of the emergency room in order to inaugurate the passage toward becoming a soon-to-be-patient. Here they establish the first contact, in a logic of openness to the other and in recognition of the suffering, through interaction rituals and relational skills. Entry into psychiatric emergency departments takes place in three characteristic stages of a rite of passage (preliminary, liminary, and post-liminary). This means that it includes a rite of separation (activation phase) in which the arrivals wish to get rid of this initial state of suffering; a rite of margin (apprehension) which attests to this transitory state of loss of reference points, anxiety and self-questioning; and finally, a rite of incorporation (appropriation) in relation to a new status. Figure 5 illustrates this phenomenon.

We wish to focus on the apprehension phase, which is representative of the waiting time in the emergency room, and which presents characteristics of liminality. This concept refers to Van Gennep’s principle of threshold, which he develops further (limens). For Turner (1990), people are, at this stage, in an in-between state, far from the norm, and dispossessed of their status. They are in a state of suspension until they get better. It is during this phase that the processes of destructuring, of questioning norms, reference points, values and identity (social, family, professional) are put in place. This is also, and above all, the moment of opening and transformation of the individual, or of a group of individuals, to lead them to a more mature modified state. In psychology, liminality is often associated with the passage from adolescence to adulthood (Jeffrey, 2008), or with the trauma clinic which places individuals in a position of liminality when the boundaries between inside and outside explode (Baubet, 2010). In our context, we often underestimate these waiting places—these liminal spaces, wrongly perceived as non-therapeutic. Their conditions (architectural, human, temporal) are often not thought through, as evidenced by the many complaints on this subject: discomfort, cold and heavy atmosphere, violence, inordinate time, etc. (Meriaux et al., 2021). The consequences are multiple and affect the patient themself, the other patients present, and the caregiver who must create a climate of trust in this already tense context. However, this dimension of being “outside,” which is fundamental to liminality, is not only to be conceived as a situation of margin to the norm and of passivity. It is precisely within this liminal space that the relationship will be played out and a new place, or even a new status, will be negotiated. All the more so since this welcome comes at a time of crisis for the individual—the crisis may be developmental or take its source in an external event that will upset the internal balance—which places the subject in a liminal space in search of a new balance. The Chinese pictogram of crisis symbolizes the dichotomous nature and the double potentiality, positive and negative, of the crisis and brings together the dimensions of danger and opportunity for change. The subject can move either toward a state of better equilibrium (transformation phase) or toward a regressive state and increase psychic and relational vulnerability (Denis and Hendrick, 2017).

The concept of rites of passage and its underlying processes has given us reference points for identifying the phenomenon of welcoming and considering its evolution over time. However, we found it useful to go beyond the tripartite nature of the model by adding an additional phase: affiliation (Figure 5). In fact, liminality thought as a waiting space is not enough to hope for a change in the individual. A third person must also intervene in this process. Through this emergent phase, we wanted to insist on the fundamental character of the relational shaping between the protagonists of the encounter. Studies on rites of passage are often restricted to a unilateral vision of the process, sometimes ignoring the interactional aspect and what is at stake in the relationship. Van Gennep’s (1909) incorporation phase gives the impression that it is the hosts who accept and integrate the newcomer into the community. It is “the house” that accepts the stranger and makes them a new part of the private space (Barazon, 2010). But what about the newcomer’s point of view? From their subjectivity? Can they refuse this gift? Can they reject the host at the cost of serious consequences? How do they negotiate the relationship? The scientific literature remains rather silent on this subject. All that is known is that this newcomer must respect the new rules that govern the “house.” To cross the threshold is to take the risk of another world and other unknown codes (Montandon, 2008).

This affiliation phase is the result of negotiations in the relationship. It is essential to cross the threshold and reach the appropriation phase. We have chosen the term “affiliation” which comes from the Latin adfiliatio (adoption) and is composed of the prefix ad-, a prefix expressing closeness, proximity, adhesion. To affiliate means for a person, or a group of persons, to join an association, a group. We can see the double meaning of this notion: to get closer and to join a system of beliefs, values and representations. The term “affiliation” was used in psychology and taken up by Minuchin (1974) to designate all of the therapist’s actions aimed at linking them to the family system. These actions are active during these moments of welcome and patients are attentive to them. We are still in this liminal space. However, this space between is not only physical, and it is not only an intrapsychic time of questioning. It is also a between-man-and-man, where the I, as Buber (2012) would say, literally offers itself to a You. This “between” exemplifies creation, movement, change, in short, the becoming of all things. For the philosopher, in the authentic relationship, no one is in a position of superiority or consent (as the welcoming of the other would suggest): each person affirms the other in an immediate and disinterested recognition. It is this recognition that the participants speak of: being recognized in their suffering and in their singularity. The challenge of the welcome is then to at least partially fill this gap, to establish a reciprocity, shaped in this space between, so that these two protagonists become partners in the pursuit of care. That is the mutual trust which is so important and decisive for our participants. As Ausloos (1995) writes, it involves the therapist’s confidence in the patient’s abilities and skills in order to allow each to work, and to move toward a more egalitarian, more human relationship. This space is the place of a shared “third subjectivity,” which belongs neither to the patient nor to the practitioner, but is created “between” them (Stern, 2003a). It is in this co-created space that the welcome takes place and where the role of this soon-to-be-patient, with all that this title implies, is negotiated.

Then, we preferred the term appropriation to integration. Asking the question of welcome is not necessarily asking the question of integration, as animal or plant welcome can be included in it (Gouirand, 2011). This is because we can welcome someone warmly without wanting to integrate them into the group or their family. Thus, the lexical and morphological components of the term “appropriation” are interesting since they literally refer to the action and effect of going somewhere, toward or in favor of the private (Damián Gómez Becerra, 2018) and its definition brings the idea of attributing something to oneself, making it one’s own. Taking ownership of one’s health care pathway is a real process.

Finally, we articulated these four phases to four dimensions specific to the study context: atmosphere, emotional and perceptual experience, intersubjective relational experience, and beliefs. Being welcomed is not limited to the diachronic aspect of the rites of passage, because at each phase, other synchronic dimensions intervene and interweave with each other, building the experience of being welcomed. Among them, we find two of Dessoy (1997) three organizing centers of human systems: the quality of the environment and the influence of representations and beliefs, which are particularly active in the emergency room. Then, two other dimensions were identified taking into account the strong emotional nature of emergency situations, and the relational and intersubjective process, which the phenomenon of welcoming involves.

### Limitations and Perspectives

The study has some limitations. Firstly, only patients referred to the crisis unit were included in the study. The results may have been potentially nuanced if we had interviewed patients who have been referred directly to a psychiatric unit—without going through the crisis unit, or patients, for whom, a return home has been proposed.

Secondly, the study is vulnerable to recall bias. The lived experience constantly escapes us, it is always part of the past. In fact, the usual speaking position is one in which the person is turned toward the other with a communicative intention. It is qualified as formal and abstract and mobilizes already constructed representations and the use of generalizing formulations. This position is easily explanatory and distanced from lived experience (Vermersch, 1994/2010). Our data only reflect what the participants are aware of doing. Indeed, we have observed that the phenomenon of welcoming remains difficult to access because it involves implicit and pre-reflective lived experiences. Husserl (1950) speaks of “pre-reflective consciousness” to say that a person permanently memorizes in a passive way what he lives, and is not conscious of everything that constitutes his experience. Moreover, welcoming mobilizes an “implicit relational knowledge” (Stern, 2003b), that is, the knowledge of what to do, think and feel in a specific relational context.

What interests us for the proceeding of this study is to be able to explore the different layers of lived experience (cognitive, emotional, sensory, egoic). The Explicitation Interview (Vermersch, 1994/2010) makes it possible to explore these different layers of lived experience and to obtain a specific and in-depth singular description of the observed phenomenon.

Let us specify that in this study, we take into account the subjectivity. From the choice of the research object to the publication of the results, any qualitative research process is based on subjectivity (Malterud, 2001). As Wolcott (1994) has pointed out, qualitative research puts the researcher back at the heart of the process, and this is the main reason for its strength. We use subjectivity as a research data. To this end, the logbook facilitates the retention of field experiences, the creation of a link between the data and the researcher, and the distancing from oneself in order to see oneself in the process of acting, thinking and conceptualizing (Baribeau, 2005).

The study sheds some light on the welcome with the emergency team (i.e., psychiatric nurses, psychiatrists, psychologists, triage nurses), from the patients’ perspectives. It is suggested that the findings are applicable also in other clinical or psychotherapeutically contexts where person in psychological suffering meet with professionals for the first time.

## Conclusion

The concepts of rite of passage and welcome seem to enrich each other in the understanding of contexts likely to facilitate—or block—the engagement and the pursuit of care.

Being welcomed is not only a ritual of welcome, it allows this often uncomfortable transitional space to be modified, and for patients to be accompanied through the identity transformations, the anguish, and the various sufferings which are not lacking in these moments of crisis.

## Data Availability Statement

The raw data supporting the conclusions of this article will be made available by the authors, without undue reservation.

## Ethics Statement

The studies involving human participants were reviewed and approved by the CHU Brugmann, Belgium. The patients/participants provided their written informed consent to participate in this study.

## Author Contributions

MM and JD contributed to conception and design of the study and wrote sections of the manuscript. MM organized the database, performed the data analysis, and wrote the first draft of the manuscript. Both authors contributed to manuscript revision, read, and approved the submitted version.

## Conflict of Interest

The authors declare that the research was conducted in the absence of any commercial or financial relationships that could be construed as a potential conflict of interest.

## Publisher’s Note

All claims expressed in this article are solely those of the authors and do not necessarily represent those of their affiliated organizations, or those of the publisher, the editors and the reviewers. Any product that may be evaluated in this article, or claim that may be made by its manufacturer, is not guaranteed or endorsed by the publisher.
